# Supplementary material for: An open-source framework for large-scale, flexible evaluation of biomedical text mining systems
Source: J Biomed Discov Collab. 2008 Jan 29;3:1. doi: 10.1186/1747-5333-3-1 (PMC2276192; doi:10.1186/1747-5333-3-1)
Supplement: Additional file 1 — Evaluation platform source code. The additional file contains the software evaluation framework discussed in this paper. The most current release can be downloaded from SourceForge.net [1]. See the accompanying README file for instructions on installation and use. Also included in the distribution is a collection of UIMA wrappers for some commonly used BioNLP tools and annotated corpora. [file 1747-5333-3-1-S1.zip › bionlp_uima_repository-1.0.1/docs/api/edu/uchsc/ccp/uima/util/opendmap/DMAPStanfordPhraseGraph.html]

DMAPStanfordPhraseGraph (Center for Computational Pharmacology Util/UIMA API)


|  |  |  |
| --- | --- | --- |
| **Overview** \| **Package** \| **Class** \| **Tree** \| **Deprecated** \| **Index** \| **Help** | |  |
| **PREV CLASS**   **NEXT CLASS** | **FRAMES**    **NO FRAMES**     **All Classes** |  |
| SUMMARY: NESTED \| FIELD \| CONSTR \| METHOD | DETAIL: FIELD \| CONSTR \| METHOD |  |


---


## edu.uchsc.ccp.uima.util.opendmap Class DMAPStanfordPhraseGraph

```
java.lang.Object
  edu.uchsc.ccp.uima.util.opendmap.DMAPStanfordPhraseGraph
```

**All Implemented Interfaces:**: edu.uchsc.ccp.opendmap.DMAPPropertyFunctionHandler

---

``` public class DMAPStanfordPhraseGraph extends Object implements edu.uchsc.ccp.opendmap.DMAPPropertyFunctionHandler ```

This class packages up a Stanford Phrase Tree as a DMAP Property Function Handler.

The goal with this class is to make syntactic constraints that the Stanford Parser
identifies available to DMAP to use as constraints between the fillers of separate
slots. For example:

```
  {c-statement} := [subject dep:x] [action head:x] [object dep:x];
```

This pattern is intended to mean that a c-statement can be recognized when DMAP sees
a [subject] an [action] and an [object]. However, in addition, the property function
constraints on [subject] and [object] says that they must be syntactically "dependent"
parts of the phrase encompassing the [action] (the head of the phrase).

The syntactic property functions "dep" and "head" only make sense in the context of
some sort of syntactic information. This StanfordPhraseGraph class defines these two
property functions and uses the information from the Stanford Parser to evaluate them.

In future, it is possible to add additional syntactic functions like "subj:x" and "obj:x"
to use more Stanford Parser information.

*Note* The main problem with this at the moment is that the Stanford Parser is
often wrong about these syntactic assignments in GeneRIFs.

**Author:**
:   R. James Firby

---

**Constructor Summary**

`DMAPStanfordPhraseGraph(org.apache.uima.jcas.JCas jcas,
boolean debug)`
  
          Create a new DMAPStanfordPhraseGraph property function handler from
the annotations the Stanford Parser has left in the UIMA CAS.


| **Method Summary** | |
| --- | --- |
| `DMAPStanfordPhraseGraph` | `clone()`             Copy this property function handler and any internal record functions. |
| `boolean` | `evalFunction(String func, String arg, edu.uchsc.ccp.opendmap.Reference reference)`             Evaluate a function. |
| `boolean` | `handles(String func)`             Check whether this property function handler wants to handle the specified function. |
| `void` | `print(PrintStream stream)`             Print a human readable version of this phrase graph. |
| `void` | `recordFunction(String func, String arg, edu.uchsc.ccp.opendmap.Reference reference)`             Record a property function constraint for later evaluation. |

**Methods inherited from class java.lang.Object**

`equals, finalize, getClass, hashCode, notify, notifyAll, toString, wait, wait, wait`

**Constructor Detail**

### DMAPStanfordPhraseGraph

```
public DMAPStanfordPhraseGraph(org.apache.uima.jcas.JCas jcas,
                               boolean debug)
```

:   Create a new DMAPStanfordPhraseGraph property function handler from
    the annotations the Stanford Parser has left in the UIMA CAS.

    **Parameters:**: `jcas` - The UIMA JCas holding the Stanford Parser annotations.: `debug` - True if debugging information should be printed.


**Method Detail**

### handles

```
public boolean handles(String func)
```

:   Check whether this property function handler wants to handle the specified function.

    :   **Specified by:**: `handles` in interface `edu.uchsc.ccp.opendmap.DMAPPropertyFunctionHandler`

---


### evalFunction

```
public boolean evalFunction(String func,
                            String arg,
                            edu.uchsc.ccp.opendmap.Reference reference)
                     throws edu.uchsc.ccp.opendmap.DMAPPropertyFunctionException
```

:   Evaluate a function. That is, check to see whether the reference is consistent with
    this function and all functions saved away from other slots. If all are satisfied then
    reference is a consistent slot filler for the slot with this property function.

    :   **Specified by:**: `evalFunction` in interface `edu.uchsc.ccp.opendmap.DMAPPropertyFunctionHandler`
    :   **Throws:**: `edu.uchsc.ccp.opendmap.DMAPPropertyFunctionException`

---


### recordFunction

```
public void recordFunction(String func,
                           String arg,
                           edu.uchsc.ccp.opendmap.Reference reference)
```

:   Record a property function constraint for later evaluation.

    :   **Specified by:**: `recordFunction` in interface `edu.uchsc.ccp.opendmap.DMAPPropertyFunctionHandler`

---


### clone

```
public DMAPStanfordPhraseGraph clone()
```

:   Copy this property function handler and any internal record functions.

    :   **Specified by:**: `clone` in interface `edu.uchsc.ccp.opendmap.DMAPPropertyFunctionHandler` **Overrides:**: `clone` in class `Object`

---


### print

```
public void print(PrintStream stream)
```

:   Print a human readable version of this phrase graph.

    :   **Parameters:**: `stream` - The stream to print to.


---


|  |  |  |
| --- | --- | --- |
| **Overview** \| **Package** \| **Class** \| **Tree** \| **Deprecated** \| **Index** \| **Help** | |  |
| **PREV CLASS**   **NEXT CLASS** | **FRAMES**    **NO FRAMES**     **All Classes** |  |
| SUMMARY: NESTED \| FIELD \| CONSTR \| METHOD | DETAIL: FIELD \| CONSTR \| METHOD |  |


---
